# 86. The Platform Trial In COVID-19 Priming and BOOsting (PICOBOO): the immunogenicity, reactogenicity, and safety of different COVID-19 vaccinations administered as a second booster (fourth dose) in AZD1222 primed individuals aged 50-< 70 years old

**DOI:** 10.1093/ofid/ofae631.023

**Published:** 2025-01-29

**Authors:** Charlie McLeod, Michael Dymock, Katie Flanagan, Magdalena Plebanski, Helen Marshall, Marie Estcourt, Ushma Wadia, Christian Tjiam, Christopher C Blyth, Kanta Subbarao, Francesca Mordant, Suellen Nicholson, Saul N Faust, Ruth Thornton, Anne McKenzie, Julie Marsh, Thomas L Snelling, Peter Richmond

**Affiliations:** Telethon Kids Institute, Perth, Western Australia, Australia; Telethon Kids Institute, Perth, Western Australia, Australia; Launceston General Hospital, Launceston, Tasmania, Australia; Royal Melbourne Institute of Technology University (RMIT), Melbourne, Victoria, Australia; University of Adelaide, Adelaide, South Australia, Australia; University of Sydney, Sydney, New South Wales, Australia; Telethon Kids Institute, Perth, Western Australia, Australia; Telethon Kids Institute, Perth, Western Australia, Australia; Wesfarmers Centre of Vaccines and Infectious Diseases, Telethon Kids Institute, Nedlands, Western Australia, Australia; WHO Collaborating Centre for Reference and Research on Influenza, Melbourne, Australia, Melbourne, Victoria, Australia; Doherty Institute, Melbourne, Victoria, Australia; University of Melbourne, Melbourne, Victoria, Australia; University of Southampton and University Hospital Southampton NHS Foundation Trust , Southampton, England, United Kingdom; Telethon Kids Institute, Perth, Western Australia, Australia; Telethon Kids Institute, Perth, Western Australia, Australia; Telethon Kids Institute, Perth, Western Australia, Australia; University of Sydney, Sydney, New South Wales, Australia; University of Western Australia School of Medicine, Perth’s Children Hospital, Nedlands, Western Australia, Australia

## Abstract

**Background:**

PICOBOO is a randomised, adaptive trial evaluating the immunogenicity, reactogenicity, and safety of COVID-19 booster strategies. Here, we report data for second boosters among individuals aged 50-< 70 years old primed with AZD1222 (50-< 70y-AZD1222) until Day (D) 84.

CONSORT diagram

**Figure:**
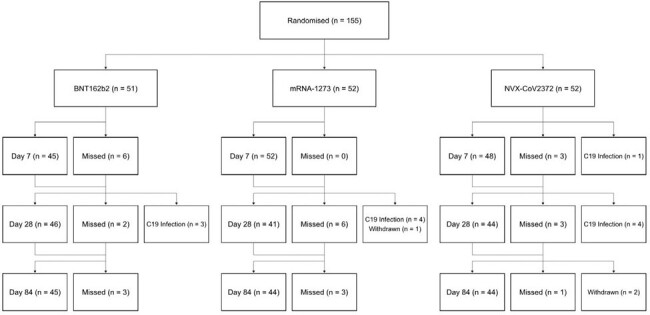

CONSORT diagram for participants recruited to the 50-<70y-AZD1222 stratum for second booster vaccines. Participants were excluded from subsequent analyses if they were infected with COVID-19 (C19 Infection) or had withdrawn. Participants that missed visits (Missed) were eligible to be included in subsequent analyses.

**Methods:**

Immunocompetent adults who received any licensed first booster at least three months prior were eligible. Participants were randomly allocated to BNT162b2, mRNA-1273 or NVX-CoV2373 1:1:1. The log_10_ concentration of anti-spike IgG was summarised as the geometric mean concentration (GMC). Reactogenicity and safety outcomes were captured. Additional analyses were performed on a subset. ACTRN12622000238774.

Baseline characteristics for study participants recruited to the 50-<70y-AZD1222 stratum for second booster vaccines summarised according to study arm.

**Figure:**
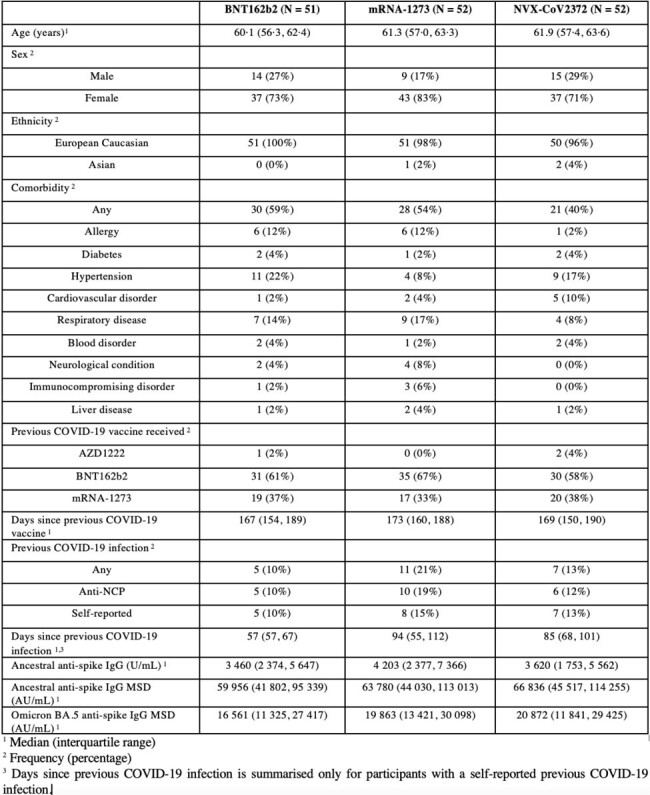

Baseline characteristics for study participants recruited to the 50-<70y-AZD1222 stratum for second booster vaccines summarised according to study arm.

**Results:**

Between 29 Mar 2022 and 2 Aug 2023, 743 participants were recruited and had D28 samples; 155 belonged to the 50-< 70y-AZD1222 stratum. At D28, the mean adjusted GMCs (95% credible intervals) were 20 690 (17 555, 23 883), 23 867 (20 144, 27 604) and 8 654 (7 267, 9 962) U/mL following boosting with BNT162b2, mRNA-1273 and NVX-CoV2372, respectively. By D84, adjusted GMCs fell to 10 976 (8 826, 13 196), 15 779 (12 512, 19 070) and 6 559 (5 220, 7 937) U/mL in each group, respectively. At D28, mean adjusted neutralisation against Ancestral virus was 159, 213 and 75 IU/mL and 100, 156 and 72 IU/mL by D84 in each group. Limited neutralisation against Omicron subvariants BA.5 and XBB.1.5 was found following boosting with all vaccines. Severe reactogenicity events were few (< 4%). Further data will be available at the time of presentation.

Posterior distributions of the anti-spike IgG adjusted GMC against Ancestral SARS-CoV-2 at D7, D28 and D84 for each study arm in participants recruited to the 50-<70y-AZD1222 stratum for second booster vaccines without COVID-19 infection after randomisation and before D28 (D7 for D7 distributions).

**Figure:**
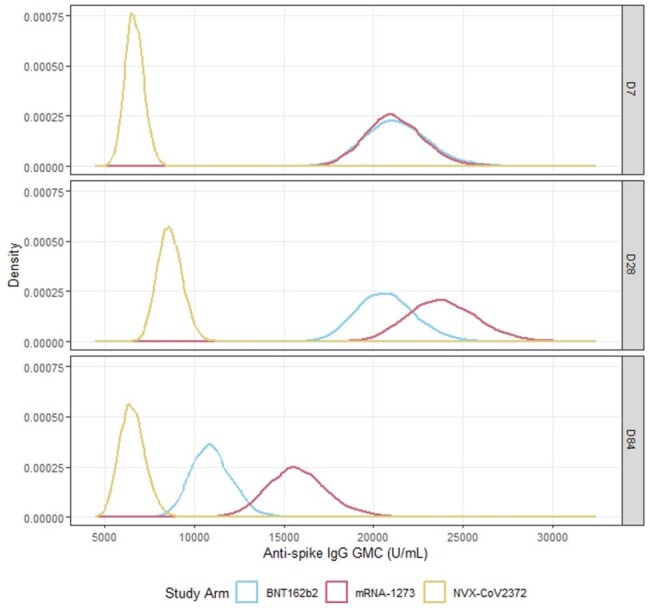

Posterior distributions of the anti-spike IgG adjusted GMC against Ancestral SARS-CoV-2 at D7, D28 and D84 for each study arm in participants recruited to the 50-<70y-AZD1222 stratum for second booster vaccines without COVID-19 infection after randomisation and before D28 (D7 for D7 distributions).

**Conclusion:**

These are the first randomised clinical trial data globally of the immunogenicity, reactogenicity and safety of second booster (fourth doses) of mRNA and protein subunit COVID-19 vaccines in adults previously primed with two doses of AZD1222. BNT162b2, mRNA-1273 and NVX-CoV2372 were well tolerated and boosted humoral immune responses. Higher binding and neutralising antibodies against Ancestral SARS-CoV-2 were observed following boosting with mRNA vaccines (BNT162b2 and mRNA-1273) compared to NVX-CoV2372 at all time points. Lower neutralising antibody responses were observed against Omicron subvariants BA.5 and XBB.1.5 following all vaccines until Day 84 highlighting the need for boosting with vaccines with greater specificity for Omicron subvariants.

Posterior distributions of the adjusted geometric mean NF50 of Ancestral SARS-CoV-2 at D28 and D84 for each study arm in study participants recruited to the 50-<70y-AZD1222 stratum for second booster vaccines without COVID-19 infection after randomisation and before D28 in the immunological subset.

**Figure:**
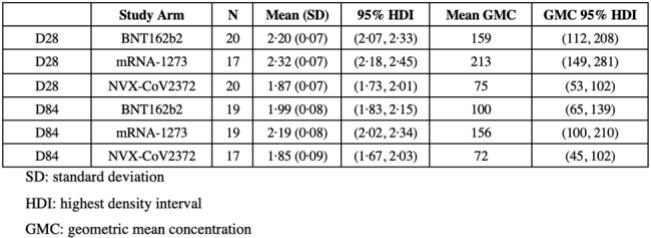

Posterior distributions of the adjusted geometric mean NF50 of Ancestral SARS-CoV-2 at D28 and D84 for each study arm in study participants recruited to the 50-<70y-AZD1222 stratum for second booster vaccines without COVID-19 infection after randomisation and before D28 in the immunological subset.

**Disclosures:**

**Magdalena Plebanski, PhD**, AstraZeneca: Grant/Research Support **Helen Marshall, MD**, ILiAD biotechnologies: Grant/Research Support **Saul N. Faust, FRCPCH PhD**, AstraZeneca: Grant/Research Support|BioNTech: Grant/Research Support|GSK: Grant/Research Support|Iliad Biotechnologies: Grant/Research Support|J&J: Grant/Research Support|J&J: Advisor, no personal payments (all honoraria paid to employing hospital)|Moderna: Grant/Research Support|Novavax: Advisor, no personal payments (all honoraria paid to employing hospital)|Pfizer: Advisor, no personal payments (all honoraria paid to employing hospital)|Sanofi: Grant/Research Support|Sanofi: Advisor, no personal payments (all honoraria paid to employing hospital)|Valneva: Grant/Research Support **Peter Richmond, MBBS**, ILiAD biotechnologies: Grant/Research Support

